# Risk Factors and Preventive Measures for Well-Leg Compartment Syndrome During Minimally Invasive Surgery in the Lithotomy Position

**DOI:** 10.3390/jcm15114213

**Published:** 2026-05-29

**Authors:** Tomoya Miura, Jun Watanabe, Shingo Tsujinaka, Yuuri Hatsuzawa, Yoh Kitamura, Kentaro Sawada, Makoto Hikage, Atsushi Mitamura, Toru Nakano, Chikashi Shibata

**Affiliations:** 1 Division of Gastroenterologic Surgery, Department of Surgery, Tohoku Medical and Pharmaceutical University, Sendai 983-8536, Miyagi, Japan; tomoyamiura@tohoku-mpu.ac.jp (T.M.);; 2 Department of Surgery, Division of Gastroenterological, General and Transplant Surgery, Jichi Medical University, Shimotsuke 329-0498, Tochigi, Japan; m06105jw@jichi.ac.jp; 3 Division of Community and Family Medicine, Jichi Medical University, Shimotsuke 329-0498, Tochigi, Japan; 4 Division of Gastroenterology, Department of Medicine and, Farncombe Family Digestive Health Research Institute, McMaster University, Hamilton, ON L8S 4K1, Canada

**Keywords:** compartment syndromes, laparoscopy, lithotomy position, monitoring, intraoperative, perioperative care, risk factors, robotic surgical procedures, well-leg compartment syndrome

## Abstract

**Background/Objectives:** Well-leg compartment syndrome is a rare but potentially life-threatening complication associated with the lithotomy position during pelvic or lower abdominal surgery. While previous studies have examined this condition in specific surgical fields, comprehensive studies focusing on minimally invasive surgery, including laparoscopic and robot-assisted surgery, have not been conducted. This scoping review aimed to summarize the latest evidence on this condition, identify risk factors, and evaluate prevention strategies. **Methods:** This scoping review was conducted according to the PRISMA-ScR guidelines. A comprehensive literature search was performed using MEDLINE, Embase, and CENTRAL. Data were extracted from studies focusing on patients who underwent minimally invasive surgery in the lithotomy position. **Results:** A total of 25 studies, including cohort studies and case reports, were included. The majority of cases were observed in procedures exceeding 4 h in duration, with a notable prevalence in the left lower extremity during gastrointestinal surgical procedures. Fasciotomy was required in the majority of reported cases. Risk factors included high body mass index, large calf circumference, prolonged operative time, peripheral vascular disease, and specific surgical positions such as head-down or head-down plus right-sided tilting. Preventive measures included intraoperative lower limb pressure monitoring, leg positioning, use of improved support devices, and reduction of operative time in the lithotomy position. **Conclusions:** This review identified key risk factors and preventive measures for compartment syndrome of the unaffected lower limb in minimally invasive pelvic surgery. However, evidence for minimally invasive surgery is limited, and standardized guidelines do not exist. Further multicenter studies are needed to establish optimal preventive measures and improve patient safety.

## 1. Introduction

Well-leg compartment syndrome (WLCS) is a rare complication associated with the lithotomy position, but it can cause lower-extremity motor and sensory deficits, and in severe cases may lead to renal dysfunction and become life-threatening. The lithotomy position is widely used in gynecologic, urologic, and colorectal surgeries to facilitate access to the perineal region; however, careful attention should be paid to the risk of WLCS [1,2,3]. In particular, minimally invasive surgery, including robot-assisted surgery for pelvic and lower abdominal diseases, may increase the risk of WLCS due to prolonged lithotomy. WLCS develops due to decreased perfusion pressure and increased tissue pressure within the lower leg compartments, resulting in neurological impairment, ischemic changes, muscle necrosis, and contractures [4,5]. If left untreated, WLCS can progress to rhabdomyolysis, myoglobinuria, acute kidney injury, and even life-threatening conditions [1]. Early diagnosis is crucial, and fasciotomy is required to relieve compartment pressure; however, this procedure may lead to long-term complications such as impaired ambulation and reduced activities of daily living [1].

Detecting symptoms in patients under general anesthesia is challenging, making preventive measures and early recognition of WLCS symptoms at each stage essential. Identifying high-risk patients and implementing perioperative risk-reduction strategies are considered essential for the prevention of WLCS. However, no consensus-based guidelines have clearly defined the risk factors for WLCS or established standardized preventive measures [6]. Although several narrative reviews have discussed WLCS [1,7], and some systematic reviews [6,8] have been conducted in specific surgical fields, no comprehensive systematic or scoping review has specifically focused on WLCS in laparoscopic or robot-assisted surgical settings.

This scoping review aimed to consolidate current knowledge on WLCS associated with minimally invasive surgery in the lithotomy position, identify gaps in the literature, and explore future research directions. The focus was on identifying risk factors and optimizing preventive strategies for WLCS. Ultimately, this review aimed to support the establishment of evidence-based guidelines to enhance surgical safety.

## 2. Materials and Methods

In developing this protocol manuscript, we referred to the PRISMA extension for scoping reviews (PRISMA-ScR) guidelines, and the PRISMA 2020 Checklist is included in the Supplementary Materials [9]. The methodological framework for the planned scoping review was based on the approach proposed by the Joanna Briggs Institute (JBI) [10]. This framework outlined five key phases to guide the review process: first, the formulation of a clear and focused research inquiry; second, a comprehensive search to locate pertinent literature; third, the application of inclusion criteria to filter eligible studies; fourth, the systematic collection and organization of relevant information; and finally, the interpretation and synthesis of the results into a coherent summary. To enhance transparency, a detailed protocol was registered on the Open Science Framework (OSF) on 26 August 2025 (Project DOI:10.17605/OSF.IO/6Z74X) and is publicly available (https://osf.io/6z74x/ (accessed on 26 May 2026)).

Stage 1: Identifying the research question

We focused on WLCS associated with the lithotomy position in pelvic surgeries, and the research questions are as follows:

1. What is the current state of evidence and clinical understanding concerning the occurrence of WLCS during surgeries performed in the lithotomy position?
2. What are the risk factors for WLCS during surgeries performed in the lithotomy position?
3. What are the effective preventive measures for WLCS during surgeries performed in the lithotomy position?

Stage 2: Identifying relevant studies

We used the Population, Concept, and Context (PCC) framework by the JBI for scoping review [10] to define the inclusion criteria as follows:

Population

All studies included patients undergoing minimally invasive surgery, including laparoscopic and robot-assisted surgery, in the lithotomy position.

Concept

This paper reviewed the existing literature on risk factors for the development of WLCS and the effectiveness and problems of preventive measures.

Context

In this study, we included patients undergoing abdominal surgery, including not only gastrointestinal surgery but also gynecologic and urologic surgery, because inclusion of these related fields was considered useful for broadening the evidence base and informing preventive strategies across pelvic and lower abdominal surgery.

We conducted a comprehensive literature search across multiple databases, including MEDLINE, Embase, and the Cochrane Central Register of Controlled Trials (CENTRAL) via the Cochrane Library, as well as clinical trial registries. While the Cochrane Library and trial registries yielded no eligible studies, this scoping review was conducted based on the search results obtained from MEDLINE and Embase. To capture ongoing or unpublished trials, we additionally explored trial registries such as the World Health Organization’s International Clinical Trials Registry Platform and ClinicalTrials.gov. Reference lists of eligible articles were also manually reviewed to identify further relevant studies. The detailed search strategy for each database is provided in Appendix A. The final literature review (citation check) was conducted on 1 December 2025.

The review encompassed a wide range of study designs, including randomized controlled trials (RCTs), non-RCTs, case reports, and case series. Studies published in languages other than English were excluded. No restriction was placed on geographic location or duration of follow-up. However, we excluded abstracts from conference proceedings and excluded review articles from the analysis.

Stage 3: Study selection

We selected studies following the Preferred Reporting Items for Systematic reviews and Meta-Analyses (PRISMA) flow diagram. We used the PCC framework, and the process was carried out independently by two researchers (T.M. and S.T.). The two authors compared their lists, and any differences in opinion were resolved by discussion and, where this failed, through arbitration by a third researcher (J.W. and C.S.).

Stage 4: Charting the data

Data extraction was carried out by one researcher (T.M.) using standard data extraction forms including author, year, country, clinical department, study type, number of participants, WLCS preventive measure, control, and outcomes. One researcher (S.T.) confirmed the data extraction. If necessary, we contacted the authors of these studies.

Stage 5: Collating, summarizing, and reporting the results

We presented the results of the search in the PRISMA flow diagram. We qualitatively organized the extracted data above.

## 3. Results

### 3.1. Selection of Resources/Search Results

A total of 206 records were initially identified through database searching. After the removal of 87 duplicate records, 119 records remained for title and abstract screening, of which 81 were excluded. Following the exclusion of 13 reports for which the full text could not be retrieved, 25 articles were assessed for full-text eligibility.

**Figure 1:**
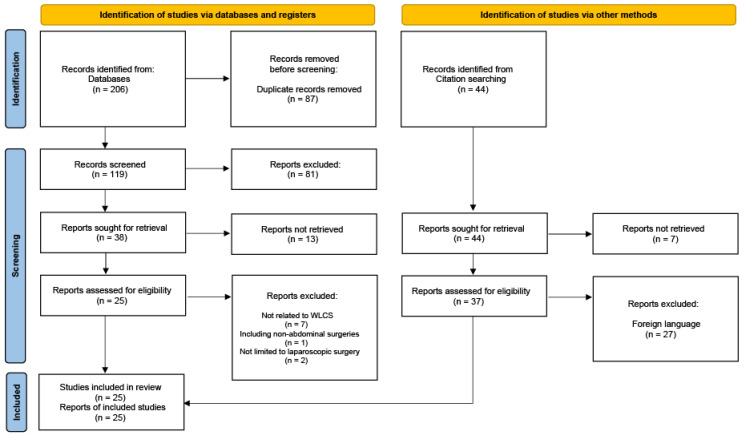
Flow diagram of the scoping review. PRISMA 2020 flow diagram for new systematic which included searches of date beses, registers and other sources.

After excluding studies that were not directly related to WLCS, those including non-abdominal procedures, and those not involving laparoscopic surgery, 15 studies met the inclusion criteria [2,3,11,12,13,14,15,16,17,18,19,20,21,22,23]. In addition, citation searching yielded 44 results. After retrieving 37 full-text articles, 27 were excluded due to being in a foreign language (all Japanese), leaving 10 relevant reports for inclusion [24,25,26,27,28,29,30,31,32,33]. Consequently, a total of 25 reports were included in this scoping review (Figure 1).

### 3.2. Characteristics of Included Studies

Of the 25 included reports, six were clinical studies, comprising three [17,18,23] prospective and three [20,22,33] retrospective studies. The remaining 19 articles were primarily case reports of WLCS (Table 1) [2,3,11,12,13,14,15,16,19,21,24,25,26,27,28,29,30,31,32]. One of these reports also included a subsequent case series with before–after comparison of a preventive measure [19].

Among the reports describing WLCS cases, nine reports originated from Japan [2,3,13,15,16,19,21,24,31], while six were conducted in Europe, including reports from the United Kingdom [14,28,33], Belgium [25], Germany [27], and Denmark [29].

Four reports investigated risk factors for WLCS (Table 2) [17,18,22,33]. These investigations were conducted as two retrospective and two prospective studies. These studies identified postoperative creatine kinase elevation [22] and increased lower limb pressure [17,18] as potential risk factors. It should be noted that, in one study, factors were evaluated for increased maximum external pressure, not for WLCS occurrence [18].

Regarding preventive strategies for WLCS, one retrospective study [20] and two prospective studies [17,23] evaluated prevention through intraoperative pressure monitoring or the use of a novel leg holder device. Additionally, one case report with subsequent before–after comparison assessed a preventive approach in rectal cancer surgery by avoiding the lithotomy position whenever feasible [19] (Table 3).

### 3.3. The Clinical Characteristics of WLCS During Laparoscopic Surgery in the Lithotomy Position (Table 1)

A total of eight WLCS reports were derived from the field of gastrointestinal surgery, whereas seven and five reports originated from gynecology and urology, respectively. The gastrointestinal surgical cases were associated with procedures performed for rectal cancer and sigmoid colon cancer. The urological cases involved surgeries for prostate cancer, bladder cancer, and ureteral cancer. Reports from gynecology comprised procedures performed for uterine fibroids, uterine malignancies, and endometriosis.

Among the included studies, only one reported the incidence of WLCS. Pridgeon et al. reported that 9 out of 3110 patients (0.29%) who underwent robot-assisted radical prostatectomy (RARP) for prostate cancer developed the condition [33].

In reports from gastrointestinal surgery, two cases were bilateral, and, except for one case where the side was not specified, all others occurred on the left side. In urology and gynecology, there was no consistent trend in laterality among surgical cases. Across all clinical departments, the operative time for all cases that developed WLCS exceeded 4 h, with the exception of one case.

The majority of WLCS cases were treated with fasciotomy. The procedure was utilized in 89% of cases in the Pridgeon et al. [33] and 80% in the report by Nishino et al. [3]. Among other case reports describing treatment outcomes, 12 of 16 patients (75%) underwent fasciotomy.

Nishino et al. reported on the sequelae of well-leg compartment syndrome (WLCS), noting that residual sensory deficits were observed in three out of eight cases (38%) who underwent fasciotomy [3]. Elsewhere in the literature, residual muscle weakness was reported in one out of 9 cases where the clinical course was documented.

### 3.4. Risk Factors of WLCS During Laparoscopic Surgery in Lithotomy Position (Table 2)

A total of four studies [17,18,22,33] investigated risk factors for WLCS in patients undergoing laparoscopic surgery in the lithotomy position. The risk factors identified in specific studies included a high body mass index (BMI) or high body weight, as well as a prolonged operative time. The BMI threshold was identified as a risk factor differed across regional cohorts. Suzuki et al. [18] used a BMI > 25 threshold (based on the Asia-Pacific obesity criteria), while the UK study by Pridgeon et al. [33] used a BMI > 30 threshold (corresponding to the WHO’s Western definition of obesity). Although absolute values varied by ethnicity, high BMI based on regional criteria was repeatedly reported as a WLCS-related factor in specific studies.

One of these studies, conducted in urology [33], analyzed the characteristics of nine WLCS cases identified from patients who underwent RARP for prostate cancer at multiple medical institutions, identifying peripheral vascular disease and an early learning curve (fewer than 20 RARP experiences) as additional risk factors.

In the three studies [17,18,22] from the field of gastrointestinal surgery, intraoperative lower extremity external pressure measurement or postoperative serum CK level measurement revealed additional physical risk factors, including male sex, age under 60, maximum left calf circumference greater than 35 cm, and preoperative calf circumference greater than 33 cm. Furthermore, high-risk surgical positions were identified as a leg elevation angle of 60° or more, head-down position (for more than 180 min), and rightward tilt of the operating table. Considering the results in Table 1 showing that WLCS is more common on the left side during gastrointestinal surgery, this suggests a possible association between tilting the operating table to the right and the development of WLCS.

### 3.5. Precautions of WLCS During Laparoscopic Surgery in Lithotomy Position (Table 3)

In this scoping review, four studies [17,19,20,23] examined preventive measures for WLCS. The preventive measures reported in these studies were intraoperative lower limb pressure monitoring, the use of new type lithotomy stirrups, and minimizing the duration of the lithotomy position.

Two studies [17,20] emphasized the importance of perioperative lower limb pressure monitoring as a preventive measure for WLCS. They employed a threshold of 30 or 50 mmHg. The timing of pressure measurement and the response after pressure measurement differ in each study.

In the study adopting a threshold of 30 mmHg, the pressure was measured every 30 min and adjusted to ≤30 mmHg. If the pressure reached ≥30 mmHg, the surgery was stopped, the leg position was released and repositioned, the pressure was reduced to ≤30 mmHg, and the procedure was resumed [20].

In the study in which a threshold of 50 mmHg was used, when pressures exceeding 50 mmHg were observed, the position of the lower leg within the lithotomy stirrups was adjusted to reduce the pressure.

In addition to lower limb pressure monitoring, one study investigated the use of modified lithotomy stirrups as a preventive strategy for WLCS [23]. The novel lithotomy stirrup is 1.3 times thicker, which is thought to distribute pressure evenly and prevent concentration of load when changing position. In the study, the novel stirrups effectively reduced lower limb pressure across various surgical positions. However, it should be noted that this study was conducted on 30 healthy volunteers rather than surgical patients, and thus its characteristics differ from those of the other clinical studies.

Regarding more fundamental preventive measures, one case report with a subsequent before–after comparison investigated minimizing the duration of the lithotomy position to only the period required for perineal maneuvers. After implementation of this approach, no further WLCS occurred, and no significant prolongation of operative time was observed [19].

## 4. Discussion

This scoping review examined 25 relevant studies to clarify the characteristics of WLCS in the lithotomy position during laparoscopic surgery. The results revealed that in almost all cases, the operative time exceeded 4 h, WLCS occurred more frequently in the left lower extremity during gastrointestinal surgery, and fasciotomy was performed in over 70% of cases. While direct evidence is currently lacking, the high frequency of WLCS in the left lower extremity might be due to the head-down tilt accompanied by rightward rotation during gastrointestinal procedures, particularly sigmoid and rectal surgeries, which could further decrease perfusion to the left leg. Reported WLCS-related factors or risk indicators included high BMI or high body weight, large calf circumference, peripheral vascular disease, prolonged operative time, leg elevation, head-down positioning, right-sided table tilt, and limited surgeon experience. This study systematically summarized WLCS in the lithotomy position during laparoscopic surgery, including robot-assisted laparoscopic surgery, as a scoping review, making it a unique study.

This review highlights lower leg circumference, head-down positioning, and right-sided table tilt as WLCS-related factors or surrogate indicators that warrant further investigation. Preventive measures for WLCS include measuring lower limb surface pressure and using the lithotomy position only when anal manipulation is necessary during surgery. Previous reviews were limited to specific clinical fields such as gastrointestinal surgery [3,19], urologic surgery [16], and gynecologic surgery [34], or included open surgery [8]. Existing literature, including studies on open surgery, identifies obesity (BMI over 25), peripheral vascular disease, lithotomy position for more than 4 h, leg elevation, and the combined use of compression stockings and intermittent pneumatic compression as high-risk patient factors for WLCS [8]. Preventive measures for WLCS include limiting continuous leg elevation to no more than 4 h and keeping the patient’s legs lower than the heart for at least 15 min every 4 h [8]. Crucially, our findings suggest that the incidence of WLCS may be higher in minimally invasive surgery (MIS) compared to open surgery. While the incidence of WLCS in open surgery has historically been reported as approximately 6 (0.011%) in 52,319 cases [35], in the studies of RARP cases included in this review, the incidence of WLCS was quite high at 0.29% [33]. Pelvic surgery utilizing the lithotomy position often requires a steep head-down tilt to displace the small bowel out of the surgical field. Furthermore, the increased intraperitoneal pressure due to pneumoperitoneum may further impair blood flow to the lower extremities. These combined effects are thought to be the cause of the high incidence, and there may be a clear difference between open surgery and minimally invasive surgery. This review confirmed the findings of existing reviews, identified new risk factors and preventive measures, and expanded the current understanding of WLCS.

This scoping review provides clinically important insights into the prevention of WLCS in laparoscopic pelvic surgery. The findings indicate that patient-related factors such as prolonged operative time; increased BMI and calf circumference; and specific intraoperative positions such as steep head-down, right-sided tilt, and leg elevation are important WLCS-related factors or risk indicators. Surgeons should exercise particular caution when surgery is expected to exceed 4 h or in patients with high-risk physical characteristics. Intraoperative leg pressure monitoring is a useful strategy, but a standard threshold has not yet been established. Maintaining pressure below approximately 20–50 mmHg, along with regular reassessment and positional adjustments, may reduce the risk. Furthermore, minimizing the duration of the lithotomy position and limiting it to only essential surgical stages appears to be a simple and effective preventive measure without significant prolongation of operative time. These findings may contribute to improving patient safety in minimally invasive surgery.

This study has several limitations. Firstly, the literature search was focused on MEDLINE, Embase, and the Cochrane Library based on a pre-registered protocol; therefore, the exclusion of databases such as Scopus or Web of Science may have resulted in the omission of some relevant technological or nursing-related perioperative literature. Additionally, only studies published in English were included, which may have led to the omission of relevant studies, such as those published in other languages or in unindexed sources. Furthermore, many of the reports extracted in this study were case reports. Also, this study is a scoping review, and no formal risk-of-bias or methodological quality appraisal was performed. Therefore, the level of evidence may be relatively low, and caution is needed when interpreting the results. In addition, there are no clear criteria for lithotomy positioning methods or diagnostic criteria for WLCS, and there is high heterogeneity in surgical techniques, patient populations, and outcomes, making quantitative analysis impossible and limiting direct comparisons between studies. Finally, regarding WLCS prevention measures, it is difficult to draw clear conclusions because there are no threshold criteria for intraoperative lower limb pressure monitoring.

## 5. Conclusions

This study comprehensively assessed the current status, risk factors, and countermeasures for WLCS in minimally invasive surgery in the lithotomy position. However, WLCS cases are few, and there are no clear criteria, such as setting appropriate thresholds for intraoperative lower limb pressure measurement. Further evidence from multicenter collaborative studies is necessary to establish optimal preventive measures and improve patient safety.

## Figures and Tables

**Table 1:** WLCS during laparoscopic surgery in lithotomy position.

Author, Year	Country	Study Type	Number of Participants (Affected Side)	Disease	Surgical Procedure	Operative Time	Treatment for WLCS	Sequelae of WLCS
Ikeya 2006 [24]	Japan	Case report	1 case (Bilateral)	Rectal cancer	Laparoscopic LAR	446 min	Fasciotomy	None
Raman, 2009 [11]	USA	Case report	1 case	Prostate cancer	Robot-assisted radical prostatectomy	300 min	Fasciotomy	Recovered without sequelae
Tomassetti, 2009 [25]	Belgium	Case report	1 case	Endometriosis	Lap resection of endometriosis	480 min	Fasciotomy	Not reported
Awab, 2011 [26]	Morocco	Case report	2 cases (left)	Rectal cancer	Lap AR	600 min 720 min	Conservative treatment	Recovered without sequelae
Lawrenz, 2011 [27]	Germany	Case report	1 case	Cervical cancer	Lap Hysterectomy	Not reported	Fasciotomy	Not reported
Pridgeon, 2013 [33]	UK	Multicenter, retrospective observational study	9 cases out of 3110 cases who underwent RARP by 17 institutions between 2004 and 2011	Prostate cancer	RARP	Console time >4 h in 8 cases	Fasciotomy (7 cases (89%))	Not reported
Kalin, 2013 [28]	UK	Case report	1 case (left)	Colovesical fistula	Lap Sigmoid colectomy	300 min	Fasciotomy	Not reported
Boesgaard-Kjer, 2013 [29]	Denmark	Case report	2 cases (left in 2 cases)	Endometriosis, Myxoma	Lap resection of endometriosis Lap myomectomy	300 min 300 min	Conservative treatment	Not reported
Stornelli, 2016 [30]	USA	Case report	1 case (bilateral)	Ectopic pregnancy	Lap salpingotomy	409 min	Fasciotomy	Recovered without sequelae
Nishino, 2018 [3]	Japan	Case report and Literature review	10 cases (left in 9 cases, bilateral in 1 case)	Rectal cancer Sigmoid colon cancer	Lap AR, ISR, APR Sigmoid colectomy	409 min (290–727)	Fasciotomy (8 cases (80%))	3 cases (38%) in fasciotomy cases Sensory dysfunction
Yamamoto, 2018 [31]	Japan	Case report	1 case (bilateral)	Ureteral cancer	Robot-assisted cystectomy	419 min	Fasciotomy	Recovered without sequelae
Zheng, 2020 [12]	China	Case report	1 case	Cervical cancer	Lap hysterectomy and pelvic lymphadenectomy	Not reported	Conservative treatment	Recovered without sequelae
Sugi, 2021 [2]	Japan	Case report	1 case	Rectal cancer	Lap LAR, pelvic lymphadenectomy and ileostomy	393 min	Fasciotomy	Recovered without sequelae
Crane 2021 [14]	UK	Case report	1 case (left)	Rectal cancer	Lap AR	360 min	Fasciotomy	Muscle weakness
Sato, 2021 [15]	Japan	Case report	1 case (left)	Uterine cancer	Lap hysterectomy	709 min	Fasciotomy	Not reported
Fukuda, 2021 [13]	Japan	Case report	1 case (right)	Bladder cancer	Robo cystectomy	481 min	Fasciotomy	Recovered without sequelae
Endo, 2022 [16]	Japan	Case report	1 case (right)	Prostate cancer	Robo prostatectomy	620 min	Not reported	Not reported
Arakawa, 2023 [19]	Japan	Case report with subsequent before–after comparison	1 case (left)	Rectal cancer	Robo LAR	384 min	Conservative treatment	Not reported
Nakayama, 2024 [21]	Japan	Case report	1 case (left)	Rectal cancer	Lap LAR	507 min	Fasciotomy	Not reported
Wang, 2024 [32]	China	Case report	1 case (bilateral)	Myxoma	Lap myomectomy	118 min	Fasciotomy	Recovered without sequelae

Lap: Laparoscopic; LAR: low anterior resection; RARP: robot-assisted radical prostatectomy; AR: anterior resection; ISR: intersphincteric resection; APR: abdominoperineal resection; Robo: robot-assisted; and WLCS: well-leg compartment syndrome.

**Table 2:** Risk factors of WLCS during laparoscopic surgery in lithotomy position.

Author, Year	Country	Study Type	Participants	Control	Risk Factors of WLCS	
					Patient-Related Factor	External Factor
Pridgeon 2013 [33]	UK	Multicenter, retrospective observational study	3110 cases who underwent RARP by 17 institutions between 2004 and 2011	Patients who did not develop WLCS	BMI > 30, peripheral vascular disease,	Console time > 4 h, early learning curve (fewer than 20 RARP experiences)
Kajitani, 2022 [17]	Japan	Prospective observational pilot study	106 patients with sigmoid colon or rectal cancer	External pressure < 50 mmHg on the lower leg	High body weight, large leg diameter	Strong leg elevation (> 60°), head-down position, and right lateral tilting of the table
Suzuki, 2023 [18]	Japan	Single-arm prospective observational study	50 patients who underwent laparoscopic surgery for colorectal cancer	Not applicable: maximum external pressure was measured in all patients	BMI > 25, age < 60 years, and maximum left calf circumference > 35 cm *	No external risk factor identified *
Kusunoki, 2024 [22]	Japan	Retrospective observational study	178 patients who underwent laparoscopic or robot-assisted surgery for colorectal cancer	CK levels ≥ 250 (n = 62) VS. CK levels < 250 (n = 116)	Male sex, preoperative calf circumference ≥ 33 cm	Rectal surgery, head-down position duration ≥ 180 min

RARP: robot-assisted radical prostatectomy; BMI: body mass index; CK: creatine kinase; and WLCS: well-leg compartment syndrome. * In the Suzuki study [18], risk factors were evaluated for increased maximum external pressure, not for WLCS occurrence.

**Table 3:** Precautions of WLCS during laparoscopic surgery in lithotomy position.

Author, Year	Country	Study Type	Participants	Control	Precautions of WLCS	Outcome
Kajitani, 2022 [17]	Japan	Prospective observational pilot study	106 patients with sigmoid colon or rectal cancer	Not applicable	Adjustment of the leg position to maintain its pressure < 50 mmHg during the perioperative period	No compartment syndrome/WLCS or peroneal nerve paralysis; postoperative lower-extremity findings included pain (3.7%), numbness (1.9%), flare (1.9%), and sensory disturbance (0.9%)
Arakawa, 2023 [19]	Japan	Case report with subsequent before–after comparison	40 cases of robot-assisted low anterior resection for rectal cancer (before, n = 17; after, n = 23)	Lithotomy throughout surgery	Starting in the supine position, and repositioning to the lithotomy position after transanal irrigation	No further WLCS occurred after preventive measure implementation, with no significant prolongation of operative time
Kondo, 2023 [20]	Japan	Retrospective observational study with propensity score matching	256 cases undergoing laparoscopic or robo gynecologic surgery	Standard care without lower-leg pressure monitoring; lithotomy position released every 3–4 h	Pressure-guided lower-leg monitoring every 30 min; leg position released or adjusted when pressure reached 30 mmHg	No WLCS in the pressure-monitoring group; postoperative CK levels were significantly lower after propensity score matching
Ochi, 2024 [23]	Japan	Prospective comparative observational study	30 healthy participants	Conventional lithotomy stirrups	Use of new type lithotomy stirrups, which are 1.3 times thicker to distribute pressure evenly and prevent concentration of load	New lithotomy stirrups significantly reduced lower-limb pressure compared with conventional stirrups; clinical WLCS occurrence was not assessed

CK: creatine kinase; Robo: robot-assisted; and WLCS: well-leg compartment syndrome.

## Data Availability

No new data were created or analyzed in this study. Data sharing is not applicable to this article.

## Supplementary Materials

The following supporting information can be downloaded at: https://www.mdpi.com/article/10.3390/jcm15114213/s1, PRISMA 2020 Checklist [36].

## Abbreviations

The following abbreviations are used in this manuscript:

WLCS	Well-leg compartment syndrome
RCT	Randomized controlled trial
PRISMA	Preferred Reporting Items for Systematic reviews and Meta-Analyses
JBI	Joanna Briggs Institute
PCC	Population, Concept, and Context
BMI	Body mass index
CK	Creatine kinase
RARP	Robot-assisted radical prostatectomy

## Appendix A

Search Strategy

MEDLINE search strategy
#1. (“well leg” [tiab]OR well-leg [tiab] OR leg [tiab])
AND “compartment syndrome*” [tiab]
#2. Lithotom* [tiab]
#3. Laparoscopy [Mesh] or laparoscop* [tiab] or celioscop* [tiab]
or peritoneoscop* [tiab] or (endoscop* [tiab] AND abdom* [tiab])
#4. “Robotic surgical procedures” [Mesh] or robot* [tiab]
#5. #2 OR #3 OR #4
#6. #1 AND #5

Embase search strategy
S1 (TI (“well leg”) OR AB (“well leg”) OR TI (“well-leg”) OR AB (“well-leg”)
OR TI (leg) OR AB (leg)) AND (TI (“compartment syndrome*”)
OR AB (“compartment syndrome*”))
S2 TI (Lithotom*) OR AB (Lithotom*)
S3 EMB.EXACT.EXPLODE (“laparoscopy”) OR TI (laparoscop*) OR AB (laparoscop*)
OR TI (celioscop*) OR AB (celioscop*) OR TI (peritoneoscop*) OR AB (peritoneoscop*)
OR ((TI (endoscop*) OR AB (endoscop*)) AND (TI (abdom*) OR AB (abdom*)))
S4 EMB.EXACT.EXPLODE (“robotic surgical procedures”) OR TI (robot*)
OR AB (robot*)
S5 S2 OR S3 OR S4
S6 S1 AND S5

## References

[1] Nester M., Borrelli J. (2022). Well Leg Compartment Syndrome: Pathophysiology, Prevention, and Treatment. J. Clin. Med..

[2] Sugi T., Owada Y., Enomoto T., Ohara Y., Akashi Y., Oda T. (2021). Well-leg compartment syndrome after laparoscopic surgery for rectal cancer: A case report. Int. J. Surg. Case Rep..

[3] Nishino M., Okano M., Kawada J., Kim Y., Yamada M., Tsujinaka T. (2018). Well-leg compartment syndrome after laparoscopic low anterior resection for lower rectal cancer in the lithotomy position: A case report. Asian J. Endosc. Surg..

[4] Chase J., Harford F., Pinzur M.S., Zussman M. (2000). Intraoperative lower extremity compartment pressures in lithotomy-positioned patients. Dis. Colon Rectum.

[5] Svendsen L.B., Flink P., Wojdemann M., Riber C., Mogensen T., Secher N.H. (1997). Muscle oxygen saturation during surgery in the lithotomy position. Clin. Physiol..

[6] Mohamedahmed A.Y., Narayanasamy S., Agrawal D., Mohamedahmed M.Y., Fadul A., Ramasamy S., Husain N., Thomas P. (2024). Lessons to Learn From 36 Cases of Well-Leg Compartment Syndrome in Colorectal Surgery: A Systematic Literature Review. Cureus.

[7] Simms M., Terry T. (2005). Well leg compartment syndrome after pelvic and perineal surgery in the lithotomy position. Postgrad. Med. J..

[8] Gill M., Fligelstone L., Keating J., Jayne D., Renton S., Shearman C., Carlson G. (2019). Avoiding, diagnosing and treating well leg compartment syndrome after pelvic surgery. J. Br. Surg..

[9] Tricco A.C., Lillie E., Zarin W., O’Brien K.K., Colquhoun H., Levac D., Moher D., Peters M.D.J., Horsley T., Weeks L. (2018). PRISMA Extension for Scoping Reviews (PRISMA-ScR): Checklist and Explanation. Ann. Intern. Med..

[10] Peter M.D.J., Godfrey C.M., Mcinerney P., Soares C.B., Khalil H., Parker D. (2015). The Joanna Briggs Institute Reviewers’ Manual 2015: Methodology for JBI Scoping Reviews.

[11] Raman S.R., Jamil Z. (2009). Well leg compartment syndrome after robotic prostatectomy: A word of caution. J. Robot. Surg..

[12] Zheng S., Zhang X., Lai J., Gao X., Wang X. (2020). Lower limb (healthy leg) compartment syndrome after the patient was in the lithotomy position during the operation: A case study. Clin. Exp. Obstet. Gynecol..

[13] Fukuda M., Kawagoe I., Kochiyama T., Ando N., Kudoh O., Satoh D., Hayashida M. (2021). Well leg compartment syndrome following robot-assisted radical cystectomy in the lithotomy position: A case report. JA Clin. Rep..

[14] Crane J., Seebah K., Morrow D., Pal A. (2021). Compartment syndrome: A rare complication following laparoscopic colorectal surgery. BMJ Case Rep..

[15] Sato H., Kotani Y., Takamatsu S., Ohta M., Shiro R., Yamamoto K., Murakami K., Matsumura N. (2021). Lower leg compartment syndrome following laparoscopic uterine malignancy surgery for uterine cancer complicated by rheumatoid arthritis: A case report and literature review. Eur. J. Gynaecol. Oncol..

[16] Endo Y., Akatsuka J., Kuwahara K., Takasaki S., Takeda H., Yanagi M., Toyama Y., Mikami H., Hamasaki T., Kondo Y. (2022). A Case of Well Leg Compartment Syndrome After Robot-assisted Laparoscopic Prostatectomy:With Review. J. Med. Investig..

[17] Kajitani R., Minami M., Kubo Y., Iwaihara H., Takishita Y., Isayama M., Ohno R., Hayashi T., Sasaki T., Matsumoto Y. (2022). Intraoperative pressure monitoring of the lower leg for preventing compression-related complications associated with the lithotomy position. Surg. Endosc..

[18] Suzuki K., Sakata M., Tatsuta K., Sugiyama K., Akai T., Suzuki Y., Kawamura T., Torii K., Morita Y., Kikuchi H. (2023). Analysis of external pressure on the left calf in the Lloyd-Davies position during colorectal surgery. Surg. Today.

[19] Arakawa K., Sako A. (2023). Well-leg compartment syndrome after robot assisted laparoscopic surgery for rectal cancer: A case report. Int. J. Surg. Case Rep..

[20] Kondo E., Kubo-Kaneda M., Mori K., Yoshida K., Nii M., Toriyabe K., Maki S., Magawa S., Okamoto K., Ikeda T. (2023). Efficacy of a portable interface pressure sensor for robotic surgery in preventing compartment syndrome. Asian J. Surg..

[21] Nakayama Y., Yamaguchi M., Inoue K., Sasaki M., Tamaki K., Hidaka M. (2024). Well-leg compartment syndrome after laparoscopic low anterior resection in the lithotomy position: A case report and literature review. J. Surg. Case Rep..

[22] Kusunoki C., Uemura M., Takeda M., Sekido Y., Hata T., Hamabe A., Ogino T., Miyoshi N., Kagawa Y., Tei M. (2024). Assessing risk factors for elevated creatine kinase levels as an indicator of compartment syndrome following laparoscopic or robot-assisted colorectal cancer surgery in the lithotomy-trendelenburg position. Surg. Endosc..

[23] Ochi T., Katsuno H., Kato H., Takagi S., Kikuchi K., Nakamura K., Endo T., Matsuo K., Yasuoka H., Nishimura A. (2024). Preliminary comparative study of lower extremity pressure measurements under the conditions using former models and new lithotomy stirrups in rectal cancer surgery. World J. Surg. Oncol..

[24] Ikeya E., Taguchi J., Ohta K., Miyazaki Y., Hashimoto O., Yagi K., Yamaguchi M., Inamura S., Makuuchi H. (2006). Compartment syndrome of bilateral lower extremities following laparoscopic surgery of rectal cancer in lithotomy position: Report of a case. Surg. Today.

[25] Tomassetti C., Meuleman C., Vanacker B., D’Hooghe T. (2009). Lower limb compartment syndrome as a complication of laparoscopic laser surgery for severe endometriosis. Fertil. Steril..

[26] Awab A., El Mansoury D., Benkabbou A., Elmoussaoui R., Elhijri A., Alilou M., Azzouzi A. (2011). Acute compartment syndrome following laparoscopic colorectal surgery. Color. Dis..

[27] Lawrenz B., Kraemer B., Wallwiener D., Witte M., Fehm T., Becker S. (2011). Lower extremity compartment syndrome after laparoscopic radical hysterectomy: Brief report of an unusual complication of laparoscopic positioning requirements. J. Minim. Invasive Gynecol..

[28] Kalin A., Hariharan V., Tudor F. (2013). Unicompartment compartment syndrome following laparascopic colonic resection. BMJ Case Rep..

[29] Boesgaard-Kjer D.H., Boesgaard-Kjer D., Kjer J.J. (2013). Well-leg compartment syndrome after gynecological laparoscopic surgery. Acta Obstet. Gynecol. Scand..

[30] Stornelli N., Wydra F.B., Mitchell J.J., Stahel P.F., Fabbri S. (2016). The dangers of lithotomy positioning in the operating room: Case report of bilateral lower extremity compartment syndrome after a 90-minutes surgical procedure. Patient Saf. Surg..

[31] Yamamoto T., Fujie A., Tanikawa H., Funayama A., Fukuda K. (2018). Bilateral well leg compartment syndrome localized in the anterior and lateral compartments following urologic surgery in lithotomy position. Case Rep. Orthop..

[32] Wang X., Zhao Z., Chen J., Zhang H. (2024). Bilateral lower extremity compartment syndrome after prolonged gynecological surgery in lithotomy position: A case report. Patient Saf. Surg..

[33] Pridgeon S., Bishop C.V., Adshead J. (2013). Lower limb compartment syndrome as a complication of robot-assisted radical prostatectomy: The UK experience. BJU Int..

[34] Bauer E.C., Koch N., Erichsen C.J., Juettner T., Rein D., Janni W., Bender H.G., Fleisch M.C. (2014). Survey of compartment syndrome of the lower extremity after gynecological operations. Langenbeck’s Arch. Surg..

[35] Warner M.E., LaMaster L.M., Thoeming A.K., Marienau M.E., Warner M.A. (2001). Compartment syndrome in surgical patients. Anesthesiology.

[36] Page M.J., McKenzie J.E., Bossuyt P.M., Boutron I., Hoffmann T.C., Mulrow C.D., Shamseer L., Tetzlaff J.M., Akl E.A., Brennan S.E. (2021). The PRISMA 2020 statement: An updated guideline for reporting systematic reviews. BMJ.

